# Maternal depression treatment in HIV (M-DEPTH): Study protocol for a cluster randomized controlled trial

**DOI:** 10.1097/MD.0000000000016329

**Published:** 2019-07-05

**Authors:** Glenn J. Wagner, Ryan K. McBain, Dickens Akena, Victoria Ngo, Janet Nakigudde, Juliet Nakku, Harriet Chemusto, Jolly Beyeza-Kashesya, Violet Gwokyalya, Laura J. Faherty, Leticia Kyohangirwe, Linda Kisaakye Nabitaka, Hafsa Lukwata, Sebastian Linnemayr, Bonnie Ghosh-Dastidar, Juliet Businge, Barbara Mukasa, Rhoda K. Wanyenze

**Affiliations:** a RAND Corporation, Santa Monica, CA; b Makerere University, Kampala, Uganda; c City University of New York Graduate School of Public Health and Health Policy, New York, NY; d Mildmay Uganda, Kampala, Uganda; e Boston University School of Medicine, Boston, MA; f Ministry of Health, Uganda.

**Keywords:** ART, depression, HIV, mental health, PMTCT, pregnancy, Uganda

## Abstract

**Introduction:**

Over one-third of human immunodeficiency virus (HIV)-infected pregnant women are clinically depressed, increasing the risk of mother-to-child transmission (MTCT) of HIV, as well as negative birth and child development outcomes. This study will evaluate the efficacy and cost-effectiveness of an evidence-based stepped care treatment model for perinatal depression (maternal depression treatment in HIV [M-DEPTH]) to improve adherence to prevention of MTCT care among HIV+ women in Uganda.

**Methods:**

Eight antenatal care (ANC) clinics in Uganda will be randomized to implement either M-DEPTH (n=4) or usual care (n=4) for perinatal depression among 400 pregnant women (n=50 per clinic) between June 2019 and August 2022. At each site, women who screen positive for potential depression will be enrolled and followed for 18 months post-delivery, assessed in 6-month intervals: baseline, within 1 month of child delivery or pregnancy termination, and months 6, 12, and 18 following delivery. Primary outcomes include adherence to the prevention of mother-to-child transmission (PMTCT) care continuum—including maternal antiretroviral therapy and infant antiretrovial prophylaxis, and maternal virologic suppression; while secondary outcomes will include infant HIV status, post-natal maternal and child health outcomes, and depression treatment uptake and response. Repeated-measures multivariable regression analyses will be conducted to compare outcomes between M-DEPTH and usual care, using 2-tailed tests and an alpha cut-off of *P* <.05. Using a micro-costing approach, the research team will relate costs to outcomes, examining the incremental cost-effectiveness ration (ICER) of M-DEPTH relative to care as usual.

**Discussion:**

This cluster randomized controlled trial will be one of the first to compare the effects of an evidence-based depression care model versus usual care on adherence to each step of the PMTCT care continuum. If determined to be efficacious and cost-effective, this study will provide a model for integrating depression care into ANC clinics and promoting adherence to PMTCT.

**Trial Registration:**

NIH Clinical Trial Registry NCT03892915 (clinicaltrials.gov).

## 1 Introduction

A package of care commonly referred to as prevention of mother-to-child transmission (PMTCT) care has been scaled up across Uganda and the larger Sub-Saharan Africa (SSA) region, in efforts to eliminate vertical transmission of the human immunodeficiency virus (HIV) virus from the infected mother to the fetus or infant.^[1]^ Yet 1 in 5 new HIV cases in Uganda still result from mother-to-child transmission.^[2]^ While recent evidence suggests that nearly all Ugandan HIV-infected pregnant women receive PMTCT care,^[2]^ about 1 in 3 do not adhere to the full PMTCT care continuum, which includes maternal pre- and post-natal antiretroviral therapy (ART), infant antiretroviral prophylaxis, periodic infant HIV testing, and exclusive breast-feeding.^[3,4]^

It is estimated that over one-third of HIV-infected pregnant women are clinically depressed,^[5–7]^ and depression is associated with poor use of and adherence to PMTCT care processes.^[8–12]^ Furthermore, perinatal depression is known to have harmful effects on birth outcomes and early child development.^[13–15]^ Yet, despite the availability of evidence-based depression treatment, depression is rarely diagnosed and treated in Ugandan antenatal care (ANC) clinics, or HIV clinics, in part due to the scarcity of mental health professionals.^[16]^ Research is needed to establish a viable depression care model in the context of PMTCT care, and to examine how depression treatment may mitigate these harmful effects on adherence to each step of the PMTCT care continuum so that optimal pregnancy and maternal and child health outcomes can be achieved.

Collaborative depression care models have been used successfully to deliver depression care in low resource settings and overcome human resource constraints,^[17–19]^ including our own INDEPTH-Uganda study of a task-shifted model of depression care in HIV clinics in Uganda.^[20,21]^ Similar depression care models have been effective for perinatal depression in the US, but have not been studied in SSA or with PMTCT care; however, there is no reason to expect that evidence-based treatment would not also be effective in this population. Ugandan ANC clinics do not provide services specific to mental health, but they do offer the Ministry of Health's Family Support Group (FSG) program for HIV-positive women, which uses group education and referrals to help women adhere to PMTCT care and manage their pregnancy.^[22,23]^ These usual care services are facilitated by nurses and trained lay persons (peer mothers), who have been shown in other studies (albeit not in the context of SSA or PMTCT care) to be able to effectively administer evidence-based depression treatments with training and supervision from mental health specialists.^[24,25]^ Depression treatment and depression alleviation have been shown to improve adherence to general HIV care processes,^[26–29]^ but such benefits have not been evaluated in the more complex PMTCT care continuum.

We are about to commence data collection for a cluster randomized controlled trial (RCT) that will evaluate the effects of an evidence-based depression care model on PMTCT care adherence, the protocol of which this paper describes in detail. The primary objective of the study is to assess whether the integrated depression care model is superior to usual care on

(1) adherence to each step of the PMTCT care continuum and maternal viral suppression (primary outcomes);
(2) prevention of infant HIV infection, and maternal and child health outcomes (secondary outcomes); and
(3) depression treatment uptake and depression alleviation (depression care processes).

We will evaluate the incremental cost-effectiveness of integrating evidence-based depression care, relative to usual care. If efficacious and cost-effective, this study will provide a model for integrating depression care into ANC clinics and promoting optimal adherence to the PMTCT care continuum and maternal and child health outcomes.

## 2 Methods

### 2.1 Study design

This is a prospective, multi-site cluster RCT to evaluate the effects of task-shifted, evidence-based depression care, relative to usual care, on adherence to each step of the PMTCT care continuum. The study will be performed at 8 ANC clinics in Uganda, with each implementing either the intervention or usual care, with an allocation ratio of 1:1, from June 2019 to August 2022. Eligible participants will be enrolled based on their attendance and pregnancy status at ANC clinics, in conjunction with a positive screen for potential depression. A schedule of trial activities is shown in Table 1.

**Table 1:**
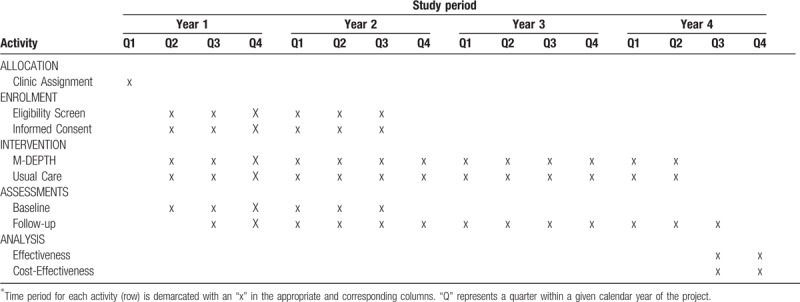
Schedule of enrolment, interventions, and assessments.

At each site, 50 HIV-positive newly pregnant women (total n = 400) who screen positive for potential depression will be enrolled and followed until 18-months post-delivery to assess how depression and depression alleviation relate to our primary (adherence to each component of the PMTCT care continuum, maternal virologic suppression) and secondary (infant HIV status; post-natal maternal and child health outcomes) outcomes, as well as processes of depression care (treatment uptake and depression alleviation among clinically depressed patients). A cost-effectiveness analysis will be used to compare the 2 study arms in relationship to these outcomes.

### 2.2 Randomization, allocation, and blinding

**Figure 1:**
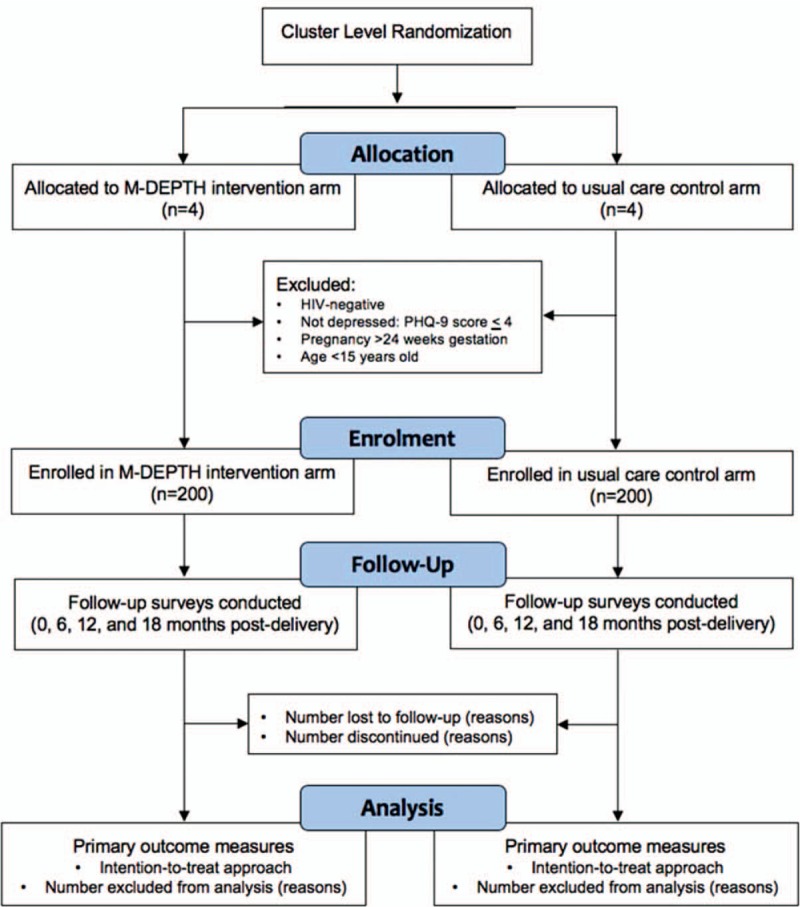
Trial flow chart ^∗^Trial flow diagram outlines the allocation, enrolment, follow-up and analysis sequence—beginning with randomization. The “allocation” sample sizes refer to the facility-level at which clustering occurs; the “enrollment” sample sizes refer to the individual patient level at which the intervention components will be administered.

The 8 ANC clinics will be randomized on a 1:1 ratio using a computer-generated list of codes and assignment. As shown in Figure 1, cluster randomization will occur before enrollment and will be stratified by level of facility [larger regional hospital and smaller health center (HCIV)], which differ primarily by size of clientele, as the ANC and mental health services are comparable, as is staff composition. To ensure balance on this factor, we will randomize clinics within these 2 strata so that 1 hospital and 3 HCIVs are assigned to both the intervention and control arms.

There is no way to blind the participants on whether or not they receive the intervention; this could potentially influence the outcomes, as clients may feel more or less incentivized to perform well in light of whether or not they receive the intervention. We do not see a way to prevent this potential bias or a way to distinguish such effects from actual intervention effects, but this limitation will be cited in reports of study findings.

### 2.3 Study setting

The study will take place at 8 antenatal care (ANC) clinics within public health facilities operated by the Uganda Ministry of Health, and which receive technical assistance in HIV reproductive health care from Mildmay Uganda. Each of the participating health facilities includes an HIV clinic, a maternal and child health center (which houses the ANC clinic as well as post-partum Mother-Baby Care clinic for HIV-infected mothers), in-patient pediatric ward, and other services. When a client of the HIV clinic becomes pregnant she is transferred to the ANC clinic (or if they first learn of their HIV diagnosis when entering the ANC clinic) to receive PMTCT and pregnancy care, including ART; she receives care within the maternal and child health center until the newborn is 18 months old, or the pregnancy is prematurely terminated, before returning to the HIV clinic for continued care. Each ANC clinic is staffed by 1 doctor-in-charge (who plays more of a supervisory role), 2 to 3 midwife nurses (who prescribe treatment), and 1 to 2 peer mothers (HIV-positive former patients who successfully managed their pregnancy and who provide volunteer assistance). The hospital ANC clinics service 400-600 HIV-positive women annually, the HCIVs 200-300. Women typically enter ANC care in the second trimester trimester, and are seen monthly during and are seen monthly during antenatal and early postpartum phases. Childbirth takes place in the in-patient maternity ward.

### 2.4 Study participants and eligibility criteria

Eligibility criteria will include:

(1) detection of pregnancy through 24 weeks gestation, to ensure at least 12 weeks remaining antenatal period for assessing adherence to all stages of PMTCT care continuum,
(2) HIV-positive serostatus and on ART for at least 4 weeks, confirmed by medical provider,
(3) age 15 years or older, and
(4) a positive screen for potential depression (Patient Health Questionnaire [PHQ]-9 >4).

At all sites, HIV-positive adult patients who are early enough in their gestation period to be eligible for the study will be informed of the study and screened for potential depression by trained peer mothers using 2 items from the Patient Health Questionnaire (PHQ-2) that assess depressed mood and loss of interest in typically pleasurable activities.

Those who screen positive (greater than zero) will receive depression psychoeducation and referred to the nurse for further depression evaluation using the full 9-item PHQ-9. Those who score >4 on the PHQ-9, signifying the potential for at least minor depression, are eligible for the study and those who express interest in participating will be connected by the peer mother or nurse to the study coordinator who will describe the study in detail, confirm eligibility and obtain written consent; these procedures are done during the same clinic visit, or the next day that the coordinator is at the clinic. Although pregnant adolescents aged 15 to 17 are eligible, these individuals are considered emancipated under Ugandan law and so parental or guardian consent will not be needed for their participation, and the adolescent will be able to provide written informed consent. We will employ the concept of youth-friendly services (i.e., using a private, confidential room to administer consent procedures; spending extra time discussing their reproductive health rights; ensuring a friendly, respectful attitude is used by the study coordinators in their interaction with youth participants).

The screening cutoffs (PHQ-2 >0, PHQ-9 >4), which are lower than what are conventionally used, are supported by a recent published psychometric evaluation of the use of the PHQ to assess depression in Uganda,^[30]^ where depressive symptoms are typically under-reported. Enrollment will be stratified so that 60% of participants enrolled at each site have PHQ-9 scores >9, signifying potential clinical depression and need for depression treatment.

### 2.5 Treatment: usual care

In Uganda, current usual care procedures for addressing depression in ANC clinics is to refer patients exhibiting significant symptoms to a mental health specialist at the District or Regional Referral hospital. There are no mental health specific services available at the ANC clinics. For HIV-positive women, each participating ANC clinic offers FSGs to provide psychosocial support, instruction and education to support pregnancy and post-partum care, including PMTCT adherence. The FSG curriculum is comprised of 24 monthly group sessions held from the antenatal phase through 18 months post-partum; each is 2 hours and devoted to a topic targeted to the stage of pregnancy. PMTCT adherence is further addressed with pill counts and adherence counseling as needed. Using usual care as the control condition does not control for the added “attention” created by the added treatment provided in the experimental arm, but it is best suited to inform policy regarding the need to augment usual care with mental health specific services.

### 2.6 Treatment: maternal depression treatment in HIV (M-DEPTH)

Drawing from evidence-based collaborative care models for depression in low resource settings,^[17,18]^ we will use the gold-standard, stepped care approach to offering psychological and pharmacologic treatment options at the 4 intervention sites. Usual care will be augmented with evidence-based problem-solving therapy (PST) through provision of manualized, individual counseling sessions, and antidepressant treatment (ADT) will be used for severe or refractory depression (or if PST is declined), which is consistent with World Health Organization (WHO) mhGAP guidelines^[31]^ for use of ADT for perinatal depression. The primary components of the depression care model are described below.

#### 2.6.1 Depression screening

All HIV-positive patients age 18 years and older who are early enough in the gestation period (≥12 gestation weeks remaining) to study eligible will be informed of the study by a peer mother and screened for potential depression using the PHQ-2 at each clinic visit while they remain eligible for the study. Patients who screen positive (scores >0) will receive depression psychoeducation from the peer mother and then referred to a midwife nurse for further depression evaluation and diagnosis.

#### 2.6.2 Depression diagnosis

Positive screens will be further evaluated by the midwife nurse using the full PHQ-9, and those with scores >4 will be further evaluated for a depressive disorder using the depression module of the Mini International Neuropsychiatric Interview (MINI)^[32]^ to diagnose Major Depression. The nurse will also assess medical stability, as depression treatment for medically unstable patients will be deferred until their condition and/or treatment is stable. Medically unstable patients will be evaluated for suicide risk and whether immediate treatment is warranted; if so, they will be referred to the site's supervising study psychiatrist for treatment.

#### 2.6.3 Psychoeducation and treatment selection

If eligible for treatment, the nurse will inform the client of the availability of PST and ADT as treatment options, in addition to encouraging the client to access usual FSG supports. Psychoeducation on treatment course, possible side effects, and benefits of treatment will be provided. Clients will have the autonomy to select their preferred treatment, but the nurse will encourage clients with mild to moderate depression (PHQ-9 <20) to consider individual PST counseling given its established efficacy and absence of significant side effect concerns, while women with severe depression (PHQ-9 ≥20) will be encouraged to consider ADT. If the client remains depressed after 8 weeks of either depression treatment (as measured by PHQ-9 at monthly visits) she will be offered to switch to or add the other form of treatment.

#### 2.6.4 Problem-solving therapy (PST)

Peer mothers will be trained to administer individual PST sessions. PST is a cognitive-behavioral intervention that trains recipients on adaptive problem-solving attitudes and the deliberate and systematic application of 4 problem-solving skills: problem definition and formulation, generation of possible solutions, selection of solutions to use, and implementation and evaluation of solutions.^[33–35]^ Clients receiving PST will attend a Core Module of 4 biweekly individual PST sessions to orient them to PST principles and methods, and learn to apply PST skills to their key stressors and barriers to PMTCT care. Additional individual monthly sessions will be provided as needed, up to 7 total sessions, for those continuing to experience depressive symptoms. Thereafter, the peer mother will have a brief “check-in” during monthly routine care visits to ask how the client is feeling in terms of mood and general mental health.

#### 2.6.5 Antidepressant therapy (ADT)

Fluoxetine, a serotonin selective reuptake inhibitor (SSRI), imipramine and amitriptyline [tricyclic antidepressants (TCAs)] are on Uganda's national formulary and freely available at the sites. ADT has potential risks for the fetus and breastfed infant, but systematic reviews of the best available research conclude that ADT is not associated with significantly increased risks, that these 3 drugs are safe in the perinatal context but need to be monitored for adverse events, and that the benefits of treatment often outweigh the risks of harm and negative impact of untreated depression.^[36–41]^ ADT will not be prescribed until after the first trimester to reduce teratogenicity risks to the infant.^[42]^ Infants will be monitored after birth for symptoms of poor neonatal adaptation syndrome, which are typically transient and managed with supportive care.^[42–45]^

Fluoxetine will typically be the first drug of choice because it is better tolerated than TCAs and has been extensively researched in the perinatal context.^[36–41]^ Starting daily dose is 20 mg of fluoxetine, 50 mg of imipramine (increasing to 75 mg after the first week), and 25 mg of amitriptyline; and the dose increments are these same dose levels for each drug. At each monthly follow-up visit, a dose increment or medication change may be considered based on measures of treatment response and side effects; in the post-partum phase, mothers will be instructed to monitor the infant for symptoms of poor neonatal adaptation (e.g., excessive crying). If side effects are tolerable, a dose or medication change is considered if no (PHQ-9 >9) or partial (PHQ-9 = 5–9) response; if side effects are intolerable, a dose/medication change is considered regardless of response, along with strategies to reduce side effects. This algorithm-based treatment decision process is repeated monthly until the patient is fully responding (PHQ-9 <5) and in remission.

#### 2.6.6 Treatment monitoring

Schedule of follow-up visits to monitor depression symptoms and treatment response will vary based on the treatment; for PST, visits will be biweekly for the first 4 sessions, and then monthly, whereas for ADT, the first 2 visits will be biweekly, and then the remainder will be monthly. A Depression Registry will be maintained by the nurses and peer mothers to record treatment data for each visit, which will facilitate future visits, supervision, and fidelity monitoring.

#### 2.6.7 Treatment discontinuation

Discontinuation will be considered if symptoms are in remission (PHQ-9 <5) for 6 months,^[46]^ and if the client is at least 6 months past completion of pregnancy, given the heightened risk of depression post-partum and in the event that there's a miscarriage or abortion. If on ADT at end of study, it will be sustained as part of usual care. Women who have a miscarriage will be transferred to the HIV clinic located at the same health facility for continued HIV care. The ANC providers and study supervisor will communicate closely with the HIV clinic staff to ensure that depression is monitored for the next 6 months, and depression and ART treatment continued or started if needed.

#### 2.6.8 Training, supervision, and fidelity

All midwife nurses (8–10 in total) and peer mothers (8 in total) at the 4 intervention sites will be trained to implement the depression care model so that related burden is balanced, and to prevent interruptions caused by staff changes. The peer mothers will be former clients who received and adhered well to the full PMTCT care continuum within the past 2 years, attended all sessions of the FSG, and expressed a desire to volunteer to mentor their peers; they will also have received a 5-day training course on co-facilitating the FSG and providing support to clients. Peer mothers will work on the 1 to 3 days per week that the clinic provides PMTCT care, and each peer mother will have a case load of no more than 10 to 12 depressed clients to administer PST. Nurses will manage 2 to 4 patients on ADT in any given month.

Supervision will be conducted one-on-one with the nurses and peer mothers and in groups by the supervisors. Supervision will be weekly for first 2 months, then biweekly for 4 months and then monthly thereafter. Supervisors will be available 24/7 for suicide or emergency consultations. Regarding fidelity, supervisors will review charts of all clients receiving ADT or PST, respectively, at each monthly supervision to assess adherence to the treatment protocol and inform the supervision. We will develop checklists for the supervisors to use to rate fidelity to each component of the protocol for PST and ADT. Fidelity measures will be incorporated at the stage of analysis as mediators of intervention effects.

### 2.7 Primary outcomes

Measures will include surveys, laboratory assays, pharmacy data, and data abstracted from medical charts and the Depression Care Registry. Survey measures that have not been translated into Luganda during prior research will be translated using standard translation, back-translation methods, and will be administered at each assessment, unless otherwise noted.

### 2.8 Adherence to PMTCT care continuum

The continuum comprises 6 components. First, maternal ART adherence will be assessed using pharmacy refill data. Dose-taking adherence (% of prescribed pills taken) will be calculated as [(pills dispensed/pills prescribed per day)/days between refills] × 100%. Self-report will measure missed doses over past 7 days, and adherence (0%–100%) over past month.^[47]^ These methods will be used to measure ADT adherence as well. Mean dose-taking adherence (% prescribed doses taken) and rate of optimal dose-taking (80+% doses taken) will be used in the primary analysis. Second, retention through antenatal and postnatal care will be assessed through chart abstraction. Participants will be classified as “retained in care” at each assessment based on whether they have been seen at the clinic in the past 2 months. For the primary analysis, successfully retained in care will be defined as retained until end of pregnancy if there was a miscarriage, or until 18 months post-partum for those who have a live birth. Third, location of delivery (health facility versus home or elsewhere) will be assessed using self-report and chart abstraction. Fourth, infant antiretroviral use and adherence will measure whether the infant was administered 6 weeks of antiretrovirals, abstracted from the mother's medical chart; adherence will be assessed via maternal self-report. Fifth, use of uniform feeding method for first 6 months after birth will be assessed via chart abstraction and maternal self-report. Sixth, infant HIV testing at week 6 and months 9 and 18 will be ascertained via chart abstraction.

### 2.9 Maternal virologic suppression

HIV viral load tests will be performed at enrollment, end of pregnancy/delivery, and month 18. Clinic staff will draw blood and store the sample; samples will be transported weekly to Mildmay by the coordinator and study driver using a standard sample carrier at a temperature of 2 to 8 C, where they will be stored at −20 C for weekly transport to a central lab (Uganda Virus Research Institute [UVRI]) for processing using the Roche Tagman assay, which is a real-time reverse transcriptase polymerase chain reaction (PCR). The UVRI lab is certified and accredited, and has served many NIH-supported HIV-related trials. The primary analysis will use a binary undetectable viral load variable at the point of pregnancy termination/delivery; viral load change from baseline will be used in secondary analyses.

### 2.10 Secondary outcomes

Secondary outcomes will focus on measures of maternal and child health. First, pregnancy outcome will be classified as live birth, stillbirth or miscarriage/abortion based on chart data, and subscales of the Peripartum Events Scale^[48]^ will be completed by the nurse—including delivery complications (5 items) and infant outcomes (12 items). Second, the mother will complete the Inventory of Functional Status After Childbirth self-report,^[49]^ as well as the New Baby subscale (9 items) of the Postpartum Adjustment Questionnaire,^[50]^ which measures the mother's relational interactions with the child. Third, the child's weight and height will be collected at each post-delivery assessment, and the mother will complete the Ages and Stages Questionnaire (ASQ-3).^[51]^ Fourth, child HIV status will be assessed at month 18, abstracted from the medical chart if an HIV test was performed as part of usual care; otherwise, the study will ask the mother to consent to the child being tested by clinic staff.

Lastly, depression care processes will be evaluated. First, the Patient Health Questionnaire (PHQ)^[52]^ will be used to measure depression symptoms (9 items). The 2 items related to sadness and loss of interest in typically pleasurable activities comprises the PHQ-2 screener. Second, depression treatment uptake and attendance in PST/ADT/FSG sessions, or other mental health services received (if referred out), will be abstracted from the client chart or Depression Care Registry. Third, depression alleviation and response to depression treatment will be defined as PHQ-9 score less than 5.^[52]^

### 2.11 Process evaluation

We will use qualitative interviews to assess provider and patient experiences of how well the treatment model is being implemented, its effects on clients and clinic operations, and the implementation barriers and strategies used to solve them. The goal is to identify key lessons learned and to ready the intervention tools for wider scale-up at end of study.

### 2.12 Provider experience

At 12-month intervals during implementation, we will interview peer mothers and midwife nurses (approximately 8 each) at the intervention sites to assess successes and challenges in screening, diagnosing and treating patients for depression; the training and supervision process; and impact of depression and its treatment on pregnancy and PMTCT care, clinic functioning, and provider job satisfaction and burnout.

### 2.13 Patient experience

A random sample of 40 participants (20 clinically depressed who were treated, and 20 not treated) will be interviewed at their final study assessment to assess their experiences with pregnancy management and the PMTCT care continuum and how depression (and treatment) influence these processes, their parenting, and quality of life; and their satisfaction with care received and competency of providers.

### 2.14 Sample size

We calculated the size of effects that this sample can detect for optimal ART adherence and undetectable viral load at completion of pregnancy, assuming attrition of 10% at pregnancy completion, 4 clusters per arm and 50 women per cluster. Based on the literature,^[53]^ we expect that 65% of the usual care group will achieve optimal (80+%) dose-taking ART adherence and 70% will achieve undetectable viral load. Our sample will have at least 80% power (alpha=0.05; 2-tailed test) to detect a 7.5 percentage point statistical difference between the 2 study arms (Cohen's δ = 0.16) for optimal adherence, and a 7 percentage point statistical difference (δ = 0.16) for undetectable viral load at end of pregnancy. To account for clustering, we calculated detectable differences using an intracluster correlation coefficient (ICC) of 0.01, which was found for maternal/perinatal health outcomes in a study conducted in 8 developing countries.^[54]^ Even if the ICC were 0.03, the effect size that can be detected for these outcomes remains similar between 8 (δ = 0.20) and 12 sites (δ = 0.17).

### 2.15 Analysis of intervention effects

Our analysis must account for clinic-level clustering. We will first compare clinic-level differences, using standard *t* tests or *t* tests weighted by cluster size in the event the observations per clinic vary. Like most cluster RCTs, we do not have the 20+ clusters needed to reliably adjust standard errors for clustering,^[55]^ so we will use the conventional approach of using regression methods on individual-level data but will conduct sensitivity analyses using a range of plausible ICC values for the outcomes.

We will use repeated-measures logistic regression to test for differences between the 2 study arms on

(I) PMTCT adherence at each stage of the PMTCT care continuum, and
(II) maternal viral suppression. Regression models will include patient characteristics, and we will explore interactions between covariates and study arm to assess which patient types are likely to be adherent and virally suppressed in depression care versus usual care.

The effect of depression care on PMTCT adherence will be examined using intention to treat as the primary approach. A secondary “completers only” analysis will be conducted, alongside ITT. In addition, we will conduct a dose-response analysis to explore the relationship between continuous implementation variables and primary outcomes. A fully specified statistical analysis plan will be written before unmasking of the data.

### 2.16 Cost and cost-effectiveness analysis

We will use a micro-costing approach to gather cost information on delivery of depression care. We will differentiate between development costs for training needed to implement the intervention, from ongoing costs of the intervention and exclude costs that are associated purely with the research activities (e.g., surveys). The evolution of the running costs will be tracked to learn whether there are cost efficiencies over time. We will also track the marginal cost of adding an additional client that will provide information for generalizability to other settings. The analysis will be performed from the clinic perspective.

We will assess whether the depression care model is cost-saving, that is, whether the increased costs for running the intervention lead to cost savings in terms of provider labor and health care costs. Costs will be estimated by tracking number of clinic visits with the nurse/peer mother, and occurrence of opportunistic infections. We will then assess whether the intervention is cost-effective by assessing the incremental cost-effectiveness ratio (ICER), defined by the difference in the per-capita costs and effects of the intervention compared to usual care. We will estimate confidence intervals for ICERs using bootstrap methods.^[56]^

### 2.17 Secondary analysis

We will conduct (bivariate analysis to initially examine significant associations between baseline depression (PHQ-9 >9) and depression alleviation over time with the primary and secondary outcome measures. Next, longitudinal analysis will be conducted to incorporate repeated observations of individuals and to explore the relationship between change in depression and outcomes over time, and to test whether depression alleviation (PHQ-9 <5) is related to change in these outcomes. Models will control for observed individual characteristics and unobservables through a random effect term to incorporate characteristics that may affect both depression and the dependent variables.

### 2.18 Ethics and dissemination

The study protocol has been reviewed and approved by Institutional Review Board at Makerere University, College of Health and Sciences, Uganda, the Uganda National Council of Science and Technology, and the RAND Corporation, Santa Monica, California. Any protocol modifications will be submitted to the IRB for review, and participants will be informed if warranted. The trial is registered with the NIH clinical trial registry (clinicaltrials.gov) and assigned the number NCT03892915. If the protocol is modified at any point, these modifications will be reflected in the NIH clinical trial registry.

We do not expect any medication-related adverse events beyond that of routine ANC and PMTCT medical care, and use of antidepressant therapy. All potential side effects will be outlined to patients during the informed consent process. Patients will be assessed and monitored with regards to psychiatric symptoms and treatment side effects by their provider on a standardized schedule using the Antidepressant Side Effect Checklist if at a site implementing the task-shifting treatment model, or as consistent with usual care at the other sites.

Given the prominence of depression in the study sample, some will express suicidal thoughts. In our prior research with HIV+ patients in Uganda, 27% of depressed patients expressed any suicidal ideation (14% had frequent thoughts) at treatment baseline. The nurses and peer mothers will be trained to implement a standardized protocol when patients report suicidal thoughts, including assessment of the severity of the ideation, intent, and means for carrying out any intent for suicide; and activation of a plan to keep the patient safe.

To ensure and maintain the scientific integrity of this human subject research project, and to protect the safety of its research participants, we will assemble a Data Safety Monitoring Board (DSMB) comprising 3 members with appropriate clinical expertise. The DSMB will have the responsibility of assuring that participants are not being exposed to unnecessary or unreasonable risks as a result of the pursuit of the study's scientific objectives. Data safety precautions to protect patient confidentiality will include lock-and key storage of any written and de-identified information, and electronic information stored on a password-protected, stored computer only accessible to members of the research team. The principal investigator and co-investigators responsible for data analysis will have access to the final dataset.

As a first step for dissemination, reporting results will be documented on ClinicalTrials.gov in accordance with NIH requirements on dissemination of clinical trial results. Information submitted will occur no later than 12 months after the primary completion date. Results produced by this investigation will be presented at international conferences and published in a timely fashion, ideally in the last year of the study period. All final peer-reviewed manuscripts that arise from this proposal will be submitted to the digital archive PubMed Central for open access. Wherever applicable, analytic code will be deposited to an appropriate public repository.

## 3 Discussion

This study will conduct a rigorous, cluster randomized controlled trial to compare the effects of an evidence-based depression care model vs. usual care on adherence to each step of the PMTCT care continuum. While there is evidence of the deleterious effects of depression on maternal and child health, and use of and adherence to PMTCT ART, this will be one of the first studies of the effects of depression on each step of the PMTCT care cascade. Using a prospective cohort of HIV+ mothers with varying levels of depression, we will collect biomarkers and other objective measures to assess how depression relates to antenatal and postpartum ART adherence, infant ART prophylaxis, uniform feeding method, HIV testing and sero-status of infant, PMTCT care retention, and maternal viral suppression.

Building on our research with task-shifted evidence-based depression care for HIV clients in Uganda, and our use of PST and ADT in low resource settings (including with pregnant women) administered by lay persons and nurses, this study may be the first to examine the efficacy and cost-effectiveness of a task-shifted, evidence-based depression care model for HIV+ pregnant women in SSA. However, given that this is evidence-based treatment, the primary innovation here is the assessment of whether depression alleviation mitigates the deleterious effects of depression on adherence to each step of the PMTCT care continuum. If efficacious and cost-effective, this study will provide a model for integrating depression care into ANC clinics and promoting optimal adherence to the PMTCT care continuum and maternal and child health outcomes.

## Author contributions

**Conceptualization:** Glenn J Wagner, Bonnie Ghosh-Dastidar, Rhoda Wanyenze, Victoria Ngo.

**Data curation:** Glenn J Wagner.

**Formal analysis:** Glenn J Wagner, Bonnie Ghosh-Dastidar.

**Funding acquisition:** Glenn J Wagner, Rhoda Wanyenze.

**Investigation:** Glenn J Wagner, Victoria Ngo, Rhoda Wanyenze.

**Methodology:** Glenn J Wagner, Dickens Akena, Victoria Ngo, Bonnie Ghosh-Dastidar, Rhoda Wanyenze, Juliet Nakku, Janet Nakigudde.

**Project administration:** Glenn J Wagner, Dickens Akena, Victoria Ngo, Rhoda Wanyenze, Harriet Chemusto, Violet Gwokyalya.

**Supervision:** Glenn J Wagner, Victoria Ngo, Dickens Akena, Rhoda Wanyenze, Violet Gwokyalya, Janet Nakigudde.

**Validation:** Glenn J Wagner.

**Visualization:** Glenn J Wagner.

**Writing – original draft:** Glenn J Wagner, Ryan K. McBain.

**Writing – review & editing:** Glenn J Wagner, Ryan K. McBain, Dickens Akena, Victoria Ngo, Janet Nakigudde, Juliet Nakku, Harriet Chemusto, Jolly Beyeza-Kashesya, Violet Gwokyalya, Laura J. Faherty, Leticia Kyohangirwe, Linda Kisaakye Nabitaka, Hafsa Lukwata, Sebastian Linnemayr, Bonnie Ghosh-Dastidar, Juliet Businge, Barbara Mukasa, Rhoda K Wanyenze.
